# Lattice-Based Logarithmic-Size Non-Interactive Deniable Ring Signatures

**DOI:** 10.3390/e23080980

**Published:** 2021-07-29

**Authors:** Huiwen Jia, Chunming Tang, Yanhua Zhang

**Affiliations:** 1 School of Mathematics and Information Science, Guangzhou University, No. 230 Wai Huan Xi Road, Guangzhou 510006, China; hwjia@gzhu.edu.cn; 2 College of Computer and Communication Engineering, Zhengzhou University of Light Industry, Zhengzhou 450002, China

**Keywords:** deniable ring signature, zero-knowledge protocols, accumulators

## Abstract

Deniable ring signature can be regarded as group signature without group manager, in which a singer is capable of singing a message anonymously, but, if necessary, each ring member is allowed to confirm or disavowal its involvement in the signature via an interactive mechanism between the ring member and the verifier. This attractive feature makes the deniable ring signature find many applications in the real world. In this work, we propose an efficient scheme with signature size logarithmic to the cardinality of the ring. From a high level, we adapt Libert et al.’s zero-knowledge argument system (Eurocrypt 2016) to allow the prover to convince the verifier that its witness satisfies an additional condition. Then, using the Fait-Shamir transformation, we get a non-interactive deniable ring signature scheme that satisfies the anonymity, traceability, and non-frameability under the small integer solution assumption in the random oracle model.

## 1. Introduction

Ring signature was first formalized by Rivest et al. [1] to deal with situations, such as leaking secrets anonymously. Specifically, a signer first picks up several public keys to form a ring; then, it generates a signature anonymously on behalf of the ring using its secret key. Any verifier is unable to get any information about the real signer, except that the message is signed by one of the ring member. This appealing feature has made the ring signature find various applications in cryptography [1,2,3]. In some situations, however, the anonymity feature is not always desirable, as it allows a user who signs a false message to shift the blame to other ring members.

It is well-known that group signature [4,5,6,7,8,9] can prevent its members from abusing anonymity, in which users are able to sign messages anonymously, but, when a dispute occurs, the group manager possessing a group master secret key is capable of revoking the anonymity of misbehaving signers. However, group signature cannot handle the leaking secrets scenario, as the the manager is always able to trace the real signer who leaks a piece of invaluable information. Besides, group signature has much higher costs on managing a dynamic group. Finally, the members are anxious that their anonymity will be or has been violated by the manager without notification.

In 2006, Komano et al. [10] formalized the notion of Deniable Ring Signature (DRS), which is as flexible as the ring signature and allows the members to confirm whether they are the real signer or not. Specifically, by using an interactive mechanism between the ring member and the verifier, it enables the real signer to confirm its signed action and allows other ring members to deny their involvement. In short, DRS can be regarded as a ‘lightweight’ group signature, i.e., group signature without the manager. For the security requirements, the DRS should satisfy:

- *Anonymity*: Any adversary should not get any information from the signature, unless the ring members are required to confirm or disavow their involvement in the signature.
- *Traceability*: Any adversary should not generate a valid ring signature such that no member will be detected as the real signer via the confirmation/disavowal protocol. In other words, the real singer cannot deny its signature.
- *Non-frameability*: Any adversary should not produce a valid ring signature such that a ring member, whose secret key is unknown to the adversary, will be detected as the real signer via the confirmation/disavowal protocol. In other words, any adversary cannot frame an honest member.

In their pioneering work, Komano et al. [10] also presented a concrete scheme under the Decisional Diffie–Hellman (DDH) assumption. However, this assumption does not hold in the quantum world [11].

### 1.1. Contributions and Technical Overview

In this work, we propose an efficient lattice-based *Non-interactive* Deniable Ring Signature (NDRS) scheme. The notion NDRS, first formalized in Reference [12], means that the confirmation or disavowal of a signature is achieved in a non-interactive manner, instead of the interactive mechanism between the ring member and the verifier. In terms of effiency, our construction is efficient in the sense that the signature size is only logarithmic to the cardinality of the ring. In the aspect of security, our construction satisfies the anonymity, traceability, and non-frameability under the Small Integer Solution (SIS) assumption in the random oracle model.

From a high level, our scheme is a natural extension of the ring signature scheme in Reference [8] to the NDRS setting. In more detail, we adapt their argument system for a tree-based accumulator [8] to allow the prover to convince the verifier that the prover knows a witness which *not only* accumulates to the root of a Merkle tree *but also* satisfies some additional conditions. Specifically, compared with the ring signature scheme in Reference [8], we add one more additional condition, an non-interactive identification scheme used by the ring members to prove their identity. Combining zero-knowledge argument systems for two or more NP relations is a general strategy widely used in previous works, such as group signatures [8,9], policy-based signatures [13], compact e-cash [14], etc.

The starting point of our construction is the Zero Knowledge Argument of Knowledge (ZKAoK) for the Merkle tree-based accumulator [8]. Specifically, the underlying hash function is defined by $h_{A}\left(x\right)=\mathrm{bin}\left(A\cdot x\;\;\;\mathrm{mod}\;q\right)\in {\left\{0,1\right\}}^{m/2}$, where the uniformly random matrix $A\in Z_{q}^{n\times m}$ serves as the common reference string, $x\in {\left\{0,1\right\}}^{m}$ is the input vector, and bin(·) denotes the coordinate-wise binary decomposition of its input. Then, by using the framework of Stern’s protocol [15], Libert et al. [8] can prove knowledge of hash chain in a zero-knowledge fashion. Besides, through the Fait-Shamir transformation, they also build ring signature with logarithmic size in the number of ring. Note that their ring signature enjoys complete anonymity. To achieve our goal that each ring member is able to generate a piece of evidence demonstrating whether it is the real signer or not, we need another matrix $B\in Z_{q}^{n\times m}$ which acts as the public key of an identification scheme. In more detail, to sign a message *M*, a signer possessing his secret x generates a zero-knowledge argument system to show that:

**Fact** **1** $d=h_{A}\left(x\right)$.
**Fact** **2** d is properly accumulated into the root of the Merkle tree.
**Fact** **3** B·x=bmodq.

We first use the procedure in Reference [8] as a sub-protocol to prove Fact 1 and Fact 2 in zero knowledge. The key point in our construction is to prove the secret in Fact 1 *simultaneously* satisfies Fact 3. To this end, we employ again the framework of Stern’s protocol [15] as a sub-protocol, such that it is compatible with the proof in Reference [8]. The details are presented in Section 3.

Then, we apply the Fait-Shamir transformation to our interactive protocol and obtain a signature scheme in the random oracle by repeating it κ=ω(logn) times to make the soundness error negligibly small. Generally, the anonymity of our NDRS scheme is based on the zero-knowledge property of the underlying argument system, while the traceability and the non-frameability are built on the fact that the underlying argument system is indeed an argument of knowledge. The description of the construction and its proof are described in Section 4.

### 1.2. Related Works

In 2006, Komano et al. [10] first introduced the notion of DRS and proposed a concrete DRS construction based on the DDH assumption. Recently, Gao et al. [12] put forward the NDRS notion, which is a direct generalization of DRS to the non-interactive setting. Besides, they proposed a concrete NDRS scheme under lattice assumptions, which is conjectured resistance against quantum computers. Their scheme, however, is shown to be insecure in Reference [16], as the scheme does not meet the ‘Traceability’ and ‘Non-frameability’ security requirements.

### 1.3. Organizations

We start in Section 2 by providing some background regarding NDRS and useful tools developed in Reference [8]. Then, in Section 3, we present an interactive protocol, which is the key component of our construction. In Section 4, we show the concrete scheme and its efficiency analysis and security proof. Finally, we conclude the paper with the obtained results.

## 2. Preliminaries

The set of integers {1,…,k} is denoted by [k]. If *S* is a finite set, x←S means that *x* is chosen uniformly at random from *S*. For b∈{0,1}, let $\bar{b}=1-b$. ⊕ denotes the bit XOR operation. For any positive integer *q*, denote by $Z_{q}$ the quotient ring Z/(qZ). Vectors, denoted by bold lowercase letters, are in column form. Matrices are represented in bold uppercase letters, and the concatenation of two matrices, say $A\in Z_{q}^{n\times m_{1}}$ and $B\in Z_{q}^{n\times m_{2}}$, is denoted by $\left[A|B\right]\in Z_{q}^{n\times (m_{1}+m_{2})}$. The tensor product is denoted by ⊗. Let $B_{2m}^{m}$ be the set of all vectors in ${\left\{0,1\right\}}^{2m}$ with Hamming weight *m*, and $S_{2m}$ be the set of all permutations of 2m elements. The abbreviation PPT means “probabilistic polynomial time”.

Throughout the paper, we denote by *n* the security parameter and define: $q=\tilde{O}\left(n\right);\;k=\left\lceil \log q\right\rceil ;\;m=2nk$. Let $G=I_{n}⊗g^{t}\in Z_{q}^{n\times nk}$, where $g^{t}$ is the row vector

$$g^{t}=\left[1\;2\;4\;\cdots \;2^{k-1}\right]\in Z_{q}^{1\times k}.$$

Note that, for any $v\in Z_{q}^{n}$, we have v=G·bin(v), where $\mathrm{bin}\left(v\right)\in {\left\{0,1\right\}}^{nk}$ denotes the binary representation of v.

### 2.1. Non-Interactive Deniable Ring Signature (NDRS)

For any positive integer N≥2, the ring *R*, formed by *N* users’ public keys, is denoted by $R=\{pk_{i_{0}},pk_{i_{1}},\ldots ,pk_{i_{N-1}}\}$. For ease of notation, we simply let $R=\{pk_{0},pk_{1},\ldots ,pk_{N-1}\}$ with ring size *N*. Now, we recall the definition and security requirements for the NDRS presented in Reference [12].

- $\mathrm{Setup}(1^{n})$: Take as input *n* and output the system parameter pp.
- KeyGen(pp): Take as input pp and output a public/secret key pair (pk,sk).
- Sign(pp,R,sk,M): Take as inputs pp, a set of *N* public keys $R=\{pk_{0},pk_{1},\ldots ,pk_{N-1}\}$, a secret key sk for which its corresponding pk∈R and a message *M* to be signed, and output a ring signature Σ.
- Verify(pp,R,M,Σ): Take as inputs pp, *R*, *M*, and Σ, and output 1 if Σ is valid or 0 otherwise.
- $\mathrm{EvidenceGen}(\mathrm{pp},R,sk_{i},\Sigma )$: Take as inputs pp, *R*, user *i*’s secret key $sk_{i}$, *M*, and Σ, and output a piece of evidence $\xi_{i}$.
- $\mathrm{EvidenckCheck}(\mathrm{pp},R,i,\xi_{i},\Sigma )$: Take as inputs pp, *R*, an identity index *i* of a user, *M* and Σ, and output “confirmation”, “disavowal”, or “reject”.

The correctness requirements for an NDRS scheme are formalized as follows:

1. The signature Σ generated by the Sign algorithm is properly accepted by the Verify algorithm, i.e., Verify(pp,R,M,Σ)=1 for any $\mathrm{pp}\leftarrow \mathrm{Setup}(1^{n})$, any (pk,sk)←KeyGen(pp), any *R* such that pk∈R and any $M\in {\left\{0,1\right\}}^{*}$.
2. The real signer of the signature Σ will generate a piece of evidence such that the evidence check algorithm outputs “confirmation”, i.e.,
  $$\mathrm{EvidenckCheck}\left(\mathrm{pp},R,i,\mathrm{EvidenceGen}(\mathrm{pp},R,sk_{i},\Sigma ),\Sigma \right)=‘‘\mathrm{confirmation}"$$
  for any valid signature Σ generated by user *i*.
3. The non-real signer should generate a piece of evidence such that the evidence check algorithm outputs “disavowal”, i.e.,
  $$\mathrm{EvidenceCheck}\left(\mathrm{pp},R,j,\mathrm{EvidenceGen}(\mathrm{pp},R,sk_{j},\Sigma ),\Sigma \right)=‘‘\mathrm{disavowal}"$$
  for any ring member j≠i.

For the security requirements, we adopt the notions and games in References [10,12]. Suppose each user has a public/private key pair supported by the Public Key Infrastructure (PKI). Let List be a public key content issued by PKI, and let MList be a list of malicious signers. Let GSet be a list of message-signature pairs generated through a challenge oracle query ${\mathrm{Ch}}_{b}\left(\cdot \right)$. An adversary is able to make the following queries.

- Add(i): on input *i*, this oracle generates a key pair ($pk_{i}$, $sk_{i}$) for user *i*, adds *i* together with the key pair to List, and returns $pk_{i}$.
- $\mathrm{Reg}(i,pk_{i})$: on inputs *i* and $pk_{i}$, this oracle registers a new signer *i* with the given public key $pk_{i}$ in List and adds user *i* to MList.
- Crpt(i): on input *i*, this oracle returns the secret key $sk_{i}$ and adds user *i* to MList.
- $\mathrm{DRSig}(i_{k};M,i_{1},\ldots ,i_{k-1},i_{k+1},\ldots ,i_{t})$: on inputs a specified user $i_{k}$, a message *M*, and a set of identities, this oracle returns a signature Σ associated with the ring formed by the input identities, by using the secret key of user $i_{k}$.
- ${\mathrm{Ch}}_{b}\left(i_{0},i_{1},M\right)$: on inputs a pair of identities $\left(i_{0},i_{1}\right)$ and *M*, this oracle returns the signature $\mathrm{Sign}(\mathrm{pp},\left\{pk_{i_{0}},pk_{i_{1}}\right\},sk_{i_{b}},M)$ for a challenge bit b←{0,1}, and adds it to GSet. This oracle is only used in the definition of anonymity.
- EGen(i,M,Σ): on inputs i,M,Σ, this oracle returns a piece of evidence demonstrating whether the entity *i* is the real signer or not. This oracle will reject the query if the input signature is an output from the challenge oracle in the experiment of anonymity.
- Hash(·): this oracle outputs a random string with a fixed length for an arbitrary input.

**Figure 1:**
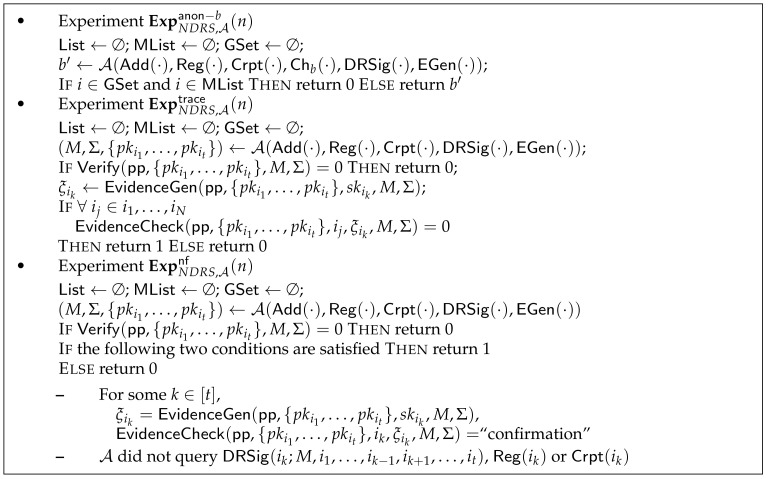
Experiments of anonymity, traceability, and non-frameability.

As mentioned before, an (N)DRS scheme should satisfy anonymity, traceability, and non-frameability. Each of these security requirements is formalized by an experiment, as shown in Figure 1.

Anonymity

For an NDRS scheme, a security parameter *n*, and a PPT adversary A, the property of anonymity is formalized using the experiment ${\mathrm{Exp}}_{NDRS,A}^{\mathrm{anon}-b}\left(n\right)$, as described in Figure 1. The advantage ${\mathrm{Adv}}_{NDRS,A}^{\mathrm{anon}}\left(n\right)$ is defined as

$${\mathrm{Adv}}_{NDRS,A}^{\mathrm{anon}}\left(n\right)=|2\mathrm{Pr}\left[{\mathrm{Exp}}_{NDRS,A}^{\mathrm{anon}-b}\left(n\right)=b\right]-1|.$$

An NDRS has anonymity if ${\mathrm{Adv}}_{NDRS,A}^{\mathrm{anon}-b}\left(n\right)$ is negligible for any PPT adversary A and security parameter *n*.

Traceability

The property of traceability is formalized using the experiment ${\mathrm{Exp}}_{NDRS,A}^{\mathrm{trace}}\left(n\right)$, as shown in Figure 1. The advantage of the adversary is given by:

$${\mathrm{Adv}}_{NDRS,A}^{\mathrm{trace}}\left(n\right)=\mathrm{Pr}\left[{\mathrm{Exp}}_{NDRS,A}^{\mathrm{trace}}\left(n\right)=1\right].$$

An NDRS is said to hold traceability if ${\mathrm{Adv}}_{NDRS,A}^{\mathrm{trace}}\left(n\right)$ is negligible for any PPT adversary A and security parameter *n*.

Non-frameability

The property of non-frameability is formalized using the experiment ${\mathrm{Exp}}_{NDRS,A}^{\mathrm{nf}}\left(n\right)$, as shown in Figure 1. The advantage of the adversary is defined as:

$${\mathrm{Adv}}_{NDRS,A}^{\mathrm{nf}}\left(n\right)=\mathrm{Pr}\left[{\mathrm{Exp}}_{NDRS,A}^{\mathrm{nf}}\left(n\right)=1\right].$$

An NDRS is said to hold non-frameability if ${\mathrm{Adv}}_{NDRS,A}^{\mathrm{nf}}\left(n\right)$ is negligible for any PPT adversary A and security parameter *n*.

### 2.2. Average-Case Lattice Problems

In this subsection, we briefly recall the average-case Small Integer Soulution (SIS) problem (in the infinity norm version) and its hardness guarantees. For more details, see References [17,18,19,20].

**Definition** **1** (Reference [17]). *Given uniformly random matrix $A\in Z_{q}^{n\times m}$, the ${\mathrm{SIS}}_{n,m,q,\beta }^{\infty }$ problem asks to find a non-zero vector $x\in Z^{m}$ such that A·x=0modq and ${∥x∥}_{\infty }\leq \beta$.*

The hardness of the SIS problem is guaranteed by a certain lattice problems in the worst case, such as the Shortest Independent Vector Problem (SIVP).

**Theorem** **1** (References [18,19,20]). *If m,β=poly(n), and $q>\beta \cdot \tilde{O}\left(\sqrt{n}\right)$, then the ${\mathrm{SIS}}_{n,m,q,\beta }^{\infty }$ problem is at least as hard as the worst-case problem ${\mathrm{SIVP}}_{\gamma }$ for some $\gamma =\beta \cdot \tilde{O}\left(\sqrt{mn}\right)$. Specifically, for $\beta =1,q=\tilde{O}\left(n\right),m=2n\left\lceil \log q\right\rceil$, the ${\mathrm{SIS}}_{n,m,q,1}^{\infty }$ problem is at least as hard as ${\mathrm{SIVP}}_{\tilde{O}\left(n\right)}$.*

### 2.3. Statistical Zero-Knowledge Argument Systems

Let $R:{\left\{0,1\right\}}^{*}\times {\left\{0,1\right\}}^{*}\to \left\{0,1\right\}$ be an NP relation. The interaction 〈P,V〉 between a prover P and a verifier V is called an interactive argument system for the relation *R* if the following two conditions hold:

Completeness

If R(x,w)=1, then

Pr[〈P(x,w),V(x)〉=1]=1.

Soundness

If R(x,w)=0, then, for every PPT $P^{*}$:

$$\mathrm{Pr}[\langle P^{*}\left(x,w\right),V\left(x\right)\rangle =1]\leq e,$$

where e∈[0,1] is called the soundness error.

In this work, we will employ the Stern-type ZKAoK [15], which is a Σ-protocol from a generalized point of view in References [21,22]. Besides, we will utilize the lattice-based string commitment scheme in Reference [23] $\mathrm{COM}:{\left\{0,1\right\}}^{*}\times {\left\{0,1\right\}}^{m/2}\to Z_{q}^{n}$, which is statistically hiding and computationally binding under the assumption that ${\mathrm{SIVP}}_{\tilde{O}\left(n\right)}$ is hard.

### 2.4. Lattice-Based Accumulator

We first recall a certain family of collision-resistant hash functions presented in Reference [8].

**Definition** **2.** *The function family $H:\;{\left\{0,1\right\}}^{nk}\times {\left\{0,1\right\}}^{nk}\to {\left\{0,1\right\}}^{nk}$ is given by $H=\{h_{A}:A\in Z_{q}^{n\times m}\}$, where*

$$h_{A}\left(u_{0},u_{1}\right)=\mathrm{bin}\left(A_{0}\cdot u_{0}+A_{1}\cdot u_{1}\;\;\;\mathrm{mod}\;q\right)\in {\left\{0,1\right\}}^{nk}.$$

*for any $\left(u_{0},u_{1}\right)\in {\left\{0,1\right\}}^{nk}\times {\left\{0,1\right\}}^{nk}$, and $A=[A_{0}|A_{1}]$ with $A_{0},A_{1}\in Z_{q}^{n\times nk}$.*

Then, we recall the Merkle tree accumulator with $N=2^{l}$ leaves based on the hash function family H above.

- $\mathrm{TSetup}(1^{n})$: On input *n*, output $\mathrm{pp}=A\leftarrow Z_{q}^{n\times m}$.
- TAcc(A,R}): Given $R={\left\{d_{j}\in {\left\{0,1\right\}}^{nk}\right\}}_{j=0}^{N-1}$, let $u_{j_{1},\ldots ,j_{l}}=d_{j}$, where $j_{1},\ldots ,j_{l}\in \left\{0,1\right\}$ is the *l* bits of *j*. Define the tree of depth *l* for the leaves $u_{0,\ldots ,0},\ldots ,u_{1,\ldots ,1}$ as follows:
  1. The nodes $u_{b_{1},\ldots ,b_{i}}$ at depth i∈[l] is given by $h_{A}\left(u_{b_{1},\ldots ,b_{i},0},u_{b_{1},\ldots ,b_{i},1}\right)$.
  2. The root $u\in {\left\{0,1\right\}}^{nk}$ is defined as $h_{A}\left(u_{0},u_{1}\right)$.
  Output u as the accumulator value.
- TWitness(A,R,d): If d∉R, output *⊥*; otherwise, ∃j∈[0,N−1] such that $d=d_{j}$. Return the witness *w* defined by:
  $$w=\left(\left(j_{1},\ldots ,j_{l}\right),\left(u_{j_{1},\ldots ,j_{l-1},\bar{j_{l}}},\ldots ,u_{j_{1},\bar{j_{2}}},u_{\bar{j_{1}}}\right)\right)\in {\left\{0,1\right\}}^{l}\times {\left({\left\{0,1\right\}}^{nk}\right)}^{l},$$
  where $u_{j_{1},\ldots ,j_{l-1},\bar{j_{l}}},\ldots ,u_{j_{1},\bar{j_{2}}},u_{\bar{j_{1}}}$ are computed by TAcc(A,R).
- TVerify(A,u,d,w): Given witness
  $$w=\left(\left(j_{1},\ldots ,j_{l}\right),\left(w_{l},\ldots ,w_{1}\right)\right)\in {\left\{0,1\right\}}^{l}\times {\left({\left\{0,1\right\}}^{nk}\right)}^{l},$$
  let $v_{l}=d$, and recursively compute $v_{l-1},\ldots ,v_{1},v_{0}\in {\left\{0,1\right\}}^{nk}$ for i∈{l−1,…,0} as follows:
  $$v_{i}=\left\{\begin{array}{cc} h_{A}\left(v_{i+1},w_{i+1}\right), & \mathrm{if}\;j_{i+1}=0;\\ h_{A}\left(w_{i+1},v_{i+1}\right), & \mathrm{if}\;j_{i+1}=1. \end{array}\right.$$
  Return 1 if $v_{0}=u$; otherwise, return 0.

In Reference [8], the authors also design an argument system for the prover P to convince the verifier V that P knows a value-witness pair (d,w) such that TVerify(A,u,d,w)=1. Toward this goal, they develop the following supporting techniques, which are necessary in our construction, as well.

- Extension of $A=[A_{0}|A_{1}]$ to $A^{*}=\left[A_{0}|0^{n\times nk}|A_{1}|0^{n\times nk}\right]\in Z_{q}^{n\times 2m}$.
- Extension of G to $G^{*}=\left[G|0^{n\times nk}\right]\in Z_{q}^{n\times m}$.
- Extensions of $v_{1},\ldots ,v_{l},w_{1},\ldots ,w_{l}$ to $v_{1}^{*},\ldots ,v_{l}^{*},w_{1}^{*},\ldots ,w_{l}^{*}\in B_{m}^{nk}$ by appending to each vector a length-nk vector with suitable Hamming weight.
- For i∈{nk,m},b∈{0,1} and $v\in {\left\{0,1\right\}}^{i}$, let ext(b,v) denote the vector $z\in {\left\{0,1\right\}}^{2i}$ of the form $z=\left(\begin{array}{c} \bar{b}\cdot v\\ b\cdot v \end{array}\right).$
- For b∈{0,1} and $\pi \in S_{m}$, define the permutation $F_{b,\pi }$ that transforms $z=\left(\begin{array}{c} z_{0}\\ z_{1} \end{array}\right)\in Z_{q}^{2m}$ consisting of 2 blocks of size *m* into $F_{b,\pi }\left(z\right)=\left(\begin{array}{c} \pi (z_{b})\\ \pi (z_{\bar{b}}) \end{array}\right)$.

Observe that, for all $c,b\in \left\{0,1\right\},\pi ,\phi \in S_{m}$ and $v,w\in {\left\{0,1\right\}}^{m}$,

$$\begin{array}{c} z=\mathrm{ext}\left(c,v\right)\wedge v\in B_{m}^{nk}⟺F_{b,\pi }\left(z\right)=\mathrm{ext}\left(c⊕b,\pi \left(v\right)\right)\wedge \pi \left(v\right)\in B_{m}^{nk};\\ y=\mathrm{ext}\left(\bar{c},w\right)\wedge w\in B_{m}^{nk}⟺F_{\bar{b},\phi }\left(y\right)=\mathrm{ext}\left(c⊕b,\phi \left(w\right)\right)\wedge \phi \left(w\right)\in B_{m}^{nk}. \end{array}$$

## 3. The Underlying Zero-Knowledge Argument System

In this section, we present an interactive protocol, upon which our NDRS scheme is built. This protocol bears much resemblance to that in Section 4.2 of Reference [8], except that one more layer is added. Specifically, in our protocol, the prover P is able to convince the verifier V on input (A,B,u,b) that P knows a secret tuple (d,w,x) such that:

- $d=h_{A}\left(x\right)\in {\left\{0,1\right\}}^{nk}$,
- TVerify(A,u,d,w)=1,
- $B\cdot x=b\;\;\;\mathrm{mod}\;q\in Z_{q}^{n}$.

More formally, the associated relation $R_{\mathrm{NDRS}}$ is given by

$$\begin{array}{c} R_{\mathrm{NDRS}}=\{(\left(A,B,u,b\right)\in Z_{q}^{n\times m}\times Z_{q}^{n\times m}\times {\left\{0,1\right\}}^{nk}\times Z_{q}^{n};\\ d\in {\left\{0,1\right\}}^{nk},w\in {\left\{0,1\right\}}^{l}\times {\left({\left\{0,1\right\}}^{nk}\right)}^{l},x\in {\left\{0,1\right\}}^{m}):\\ A\cdot x=G\cdot d\;\;\;\mathrm{mod}\;q\;\wedge \;B\cdot x=b\;\;\;\mathrm{mod}\;q\;\wedge \;\mathrm{TVerify}(A,u,d,w)=1\}. \end{array}$$

### 3.1. Description of the Interactive Protocol

The public parameters are $n,m,q,k,l,G,G^{*},\hat{A}$, and $\hat{B}$, where

$$\hat{A}=\left[A|0^{n\times m}\right],\hat{B}=\left[B|0^{n\times m}\right]\in Z_{q}^{n\times 2m}.$$

The prover P, using its witness, prepares, according to Section 2.4, the following vectors:

$$v_{i}^{*},\;w_{i}^{*}\in B_{m}^{nk},\;\;z_{i}=\mathrm{ext}\left(j_{i},v_{i}^{*}\right),\;\;y_{i}=\mathrm{ext}\left({\bar{j}}_{i},w_{i}^{*}\right),$$

for all i∈[l] such that

(1) $$\left\{\begin{array}{cc} A^{*}\cdot z_{1}+A^{*}\cdot y_{1}=G\cdot u\;\;\;\mathrm{mod}\;q; & \\ A^{*}\cdot z_{i+1}+A^{*}\cdot y_{i+1}=G^{*}\cdot v_{i}^{*}\;\;\;\mathrm{mod}\;q \end{array}\right.,$$

for all i∈[l−1]. Observe that $G^{*}\cdot v_{l}^{*}=G\cdot d$. First, P extends x into $x^{*}\in B_{2m}^{m}$. Clearly, $\hat{A}\cdot x^{*}=A\cdot x$ and $\hat{B}\cdot x^{*}=B\cdot x$. In Stern’s framework, a random permutation $\tau \leftarrow S_{2m}$ and a random ‘mask’ $r_{x}\leftarrow Z_{q}^{2m}$ give a ZKAoK of the secret x according to the equivalence $x^{*}\in B_{2m}^{m}\Leftrightarrow \tau \left(x^{*}\right)\in B_{2m}^{m}$.

After these preparations, P’s goal is to convince V that it knows the vectors $v_{i}^{*},\;w_{i}^{*}$, $z_{i},\;y_{i}$ for all i∈[l] and $x^{*}\in B_{2m}$ such that:

- Equation (1) holds;
- $\hat{A}\cdot x^{*}=G^{*}\cdot v_{l}^{*}\;\;\;\mathrm{mod}\;q$ and $\hat{B}\cdot x^{*}=b\;\;\;\mathrm{mod}\;q$.

The interaction between P and V is detailed as follows.

1. **Commitment.**P firstly picks the following randomness:
  $$\begin{array}{c} \rho_{1},\rho_{2},\rho_{3}\in {\left\{0,1\right\}}^{m/2}\;\;\mathrm{for}\;\;\mathrm{COM}\\ \tau \leftarrow S_{2m};\;b_{1},\ldots ,b_{l}\leftarrow \left\{0,1\right\};\;\pi_{1},\ldots ,\pi_{l},\;\phi_{1},\ldots ,\phi_{l}\leftarrow S_{m}\\ r_{x}\leftarrow Z_{q}^{2m};\;r_{v_{1}},\ldots ,r_{v_{l}}\leftarrow Z_{q}^{m};\;r_{z_{1}},\ldots ,r_{z_{l}},\;r_{y_{1}},\ldots ,r_{y_{l}}\leftarrow Z_{q}^{2m}. \end{array}$$
  Then, the commitment CMT=($C_{1},C_{2},C_{3}$) is sent to V, where
  $$\begin{array}{cc} C_{1} & =\mathrm{COM}(\tau ;\;\hat{A}\cdot r_{x}-G^{*}\cdot r_{v_{l}};\;\hat{B}\cdot r_{x};\;{\left\{b_{i},\pi_{i},\phi_{i}\right\}}_{i=1}^{l};\;A^{*}\cdot r_{z_{1}}+A^{*}\cdot r_{y_{1}};\\ \; & \;\;\;\;\;\;\;\;\;\;\;\;\;\;{\left\{A^{*}\cdot r_{z_{i+1}}+A^{*}\cdot r_{y_{i+1}}-G^{*}\cdot r_{v_{i}}\right\}}_{i=1}^{l-1};\;\rho_{1})\\ C_{2} & =\mathrm{COM}\left(\tau \left(r_{x}\right);\;{\left\{\pi_{i}\left(r_{v_{i}}\right),F_{b_{i},\pi_{i}}\left(r_{z_{i}}\right),F_{{\bar{b}}_{i},\phi_{i}}\left(r_{y_{i}}\right)\right\}}_{i=1}^{l};\;\rho_{2}\right)\\ C_{3} & =\mathrm{COM}\left(\tau \left(x^{*}+r_{x}\right);\;{\left\{\pi_{i}\left(v_{i}^{*}+r_{v_{i}}\right),F_{b_{i},\pi_{i}}\left(z_{i}+r_{z_{i}}\right),F_{{\bar{b}}_{i},\phi_{i}}\left(y_{i}+r_{y_{i}}\right)\right\}}_{i=1}^{l};\;\rho_{3}\right). \end{array}$$
2. **Challenge.**V sends to P a challenge Ch←{1,2,3}.
3. **Response.**P sends the response RSP depending on Ch as follows:
  - Ch=1: Let ${\tilde{x}}^{*}=\tau \left(x^{*}\right),{\tilde{r}}_{x}=\tau \left(r_{x}\right)$, and, for each i∈[l], let:
    $$\begin{array}{c} {\tilde{b}}_{i}=j_{i}⊕b_{i};\;{\tilde{v}}_{i}^{*}=\pi_{i}\left(v_{i}^{*}\right);\;{\tilde{w}}_{i}^{*}=\phi_{i}\left(w_{i}^{*}\right)\\ {\tilde{r}}_{v_{i}}=\pi_{i}\left(r_{v_{i}}\right);\;{\tilde{r}}_{z_{i}}=F_{b_{i},\pi_{i}}\left(r_{z_{i}}\right);\;{\tilde{r}}_{y_{i}}=F_{{\bar{b}}_{i},\phi_{i}}\left(r_{y_{i}}\right). \end{array}$$
    Set RSP=$\left({\tilde{x}}^{*};\;{\tilde{r}}_{x};\;{\left\{{\tilde{b}}_{i},{\tilde{v}}_{i}^{*},{\tilde{w}}_{i}^{*},{\tilde{r}}_{v_{i}},{\tilde{r}}_{z_{i}},{\tilde{r}}_{y_{i}}\right\}}_{i=1}^{l};\;\rho_{2};\;\rho_{3}\right)$.
  - Ch=2: Let $\tau^{\prime }=\tau ,\;s_{x}=x^{*}+r_{x}$, and, for each i∈[l], let:
    $$\begin{array}{c} b_{i}^{\prime }=b_{i};\;\pi_{i}^{\prime }=\pi_{i};\;\phi_{i}^{\prime }=\phi_{i};\;\\ s_{v_{i}}=v_{i}^{*}+r_{v_{i}};\;s_{z_{i}}=z_{i}+r_{z_{i}};\;s_{y_{i}}=y_{i}+r_{y_{i}}. \end{array}$$
    Set RSP=$\left(\tau^{\prime };\;s_{x};\;{\left\{b_{i}^{\prime },\pi_{i}^{\prime },\phi_{i}^{\prime },s_{v_{i}},s_{z_{i}},s_{y_{i}}\right\}}_{i=1}^{l};\;\rho_{1};\;\rho_{3}\right)$.
  - Ch=3: Let $\tau^{\prime \prime }=\tau ,\;r_{x}^{\prime }=r_{x}$, and, for each i∈[l], let:
    $$\begin{array}{c} b_{i}^{\prime \prime }=b_{i};\;\pi_{i}^{\prime \prime }=\pi_{i};\;\phi_{i}^{\prime \prime }=\phi_{i};\;\\ r_{v_{i}}^{\prime }=r_{v_{i}};\;r_{z_{i}}^{\prime }=r_{z_{i}};\;r_{y_{i}}^{\prime }=r_{y_{i}}. \end{array}$$
    Set RSP=$\left(\tau^{\prime \prime };\;r_{x}^{\prime };\;{\left\{b_{i}^{\prime \prime },\pi_{i}^{\prime \prime },\;\phi_{i}^{\prime \prime },r_{v_{i}}^{\prime },r_{z_{i}}^{\prime },r_{y_{i}}^{\prime }\right\}}_{i=1}^{l};\;\rho_{1};\;\rho_{2}\right)$.
4. **Verification.** Given RSP, V proceeds as follows.
  - Ch=1: Check that ${\tilde{x}}^{*}\in B_{2m}$ for i∈[p], ${\tilde{v}}_{i}^{*},{\tilde{w}}_{i}^{*}\in B_{m}^{nk}$ for i∈[l] and that
    $$\begin{array}{cc} C_{2} & =\mathrm{COM}\left({\tilde{r}}_{x};\;{\left\{{\tilde{r}}_{v_{i}},{\tilde{r}}_{z_{i}},{\tilde{r}}_{y_{i}}\right\}}_{i=1}^{l};\;\rho_{2}\right),\\ C_{3} & =\mathrm{COM}\left({\tilde{x}}^{*}+{\tilde{r}}_{x};\;{\left\{{\tilde{v}}_{i}^{*}+{\tilde{r}}_{v_{i}},\mathrm{ext}\left({\tilde{b}}_{i},{\tilde{v}}_{i}^{*}\right)+{\tilde{r}}_{z_{i}},\mathrm{ext}\left({\tilde{b}}_{i},{\tilde{w}}_{i}^{*}\right)+{\tilde{r}}_{y_{i}}\right\}}_{i=1}^{l};\;\rho_{3}\right). \end{array}$$
  - Ch=2: Check that
    $$\begin{array}{cc} C_{1} & =\mathrm{COM}(\tau^{\prime };\;\hat{A}\cdot s_{x}-G^{*}\cdot s_{v_{l}};\;\hat{B}\cdot s_{x}-b;\;{\left\{b_{i}^{\prime },\pi_{i}^{\prime },\phi_{i}^{\prime }\right\}}_{i=1}^{l};\;\\ \; & \;\;\;\;\;\;\;A^{*}\cdot s_{z_{1}}+A^{*}\cdot s_{y_{1}}-G\cdot u;{\left\{A^{*}\cdot s_{z_{i+1}}+A^{*}\cdot s_{y_{i+1}}-G^{*}\cdot s_{v_{i}}\right\}}_{i=1}^{l-1};\;\rho_{1}),\\ C_{3} & =\mathrm{COM}\left(\tau^{\prime }\left(s_{x}\right);\;{\left\{\pi_{i}^{\prime }\left(s_{v_{i}}\right),F_{b_{i}^{\prime },\pi_{i}^{\prime }}\left(s_{z_{i}}\right),F_{{\bar{b}}_{i}^{\prime },\phi_{i}^{\prime }}\left(s_{y_{i}}\right)\right\}}_{i=1}^{l};\;\rho_{3}\right). \end{array}$$
  - Ch=3: Check that
    $$\begin{array}{cc} C_{1} & =\mathrm{COM}(\tau^{\prime \prime };\;\hat{A}\cdot r_{x}^{\prime }-G^{*}\cdot r_{v_{l}}^{\prime };\;\hat{B}\cdot r_{x}^{\prime };\;{\left\{b_{i}^{\prime \prime },\pi_{i}^{\prime \prime },\phi_{i}^{\prime \prime }\right\}}_{i=1}^{l};\;A^{*}\cdot r_{z_{1}}^{\prime }+A^{*}\cdot r_{y_{1}}^{\prime };\\ \; & \;\;\;\;\;\;\;\;\;\;\;\;\;\;{\left\{A^{*}\cdot r_{z_{i+1}}^{\prime }+A^{*}\cdot r_{y_{i+1}}^{\prime }-G^{*}\cdot r_{v_{i}}^{\prime }\right\}}_{i=1}^{l-1};\;\rho_{1}),\\ C_{2} & =\mathrm{COM}\left(\tau^{\prime \prime }\left(r_{x}^{\prime }\right);\;{\left\{\pi_{i}^{\prime \prime }\left(r_{v_{i}}^{\prime }\right),F_{b_{i}^{\prime \prime },\pi_{i}^{\prime \prime }}\left(r_{z_{i}}^{\prime }\right),F_{{\bar{b}}_{i}^{\prime \prime },\phi_{i}^{\prime \prime }}\left(r_{y_{i}}^{\prime }\right)\right\}}_{i=1}^{l};\;\rho_{2}\right). \end{array}$$
  V outputs 1 only if all the conditions hold in each cases. Otherwise, output 0.

### 3.2. Analysis of the Interactive Protocol

We summarize several properties of the above protocol in the following theorem. Since the proof of the properties of the protocol is similar with that of Reference [8], we omit the details. (See Appendix A)

**Theorem** **2.** *The given interactive protocol has perfect completeness and communication cost $\tilde{O}\left(l\cdot n\right)$. If COM is a statistically hiding and computationally binding string commitment scheme, then it is an ZKAoK for the relation $R_{\mathrm{NDRS}}$.*

## 4. Our Non-Interactive Deniable Ring Signature Scheme from Lattices

We now construct an NDRS scheme for rings with $N=2^{l}$ users (It can be easily adapted for any other values of *N* as in Reference [8].) and prove that our construction satisfies the security requirements: anonymity, traceability, and non-frameability. We use a public Pseudo-random Generator (PRG), and a random oracle $H_{\mathrm{FS}}:\;{\left\{0,1\right\}}^{*}\to {\left\{1,2,3\right\}}^{\kappa }$.

- $\mathrm{Setup}(1^{n})$: On input *n*, output $\mathrm{pp}=A\leftarrow Z_{q}^{n\times m}$.
- KeyGen(pp): On input pp, output (pk,sk)=(d,x), where $x\leftarrow {\left\{0,1\right\}}^{m}$, and d=bin(A·xmodq).
- Sign(pp,R,sk,M): On inputs pp, $R=\{d_{0},\ldots ,d_{N-1}\}$, sk, and *M*, it works as follows (Notice that, for the public key pk corresponding to the input sk, we have pk∈R.) to output the signature Σ.
  1. Run TAcc(A,R) and obtain $u\in {\left\{0,1\right\}}^{nk}$. Recall that u is the root of the Merkle tree defined on *R*.
  2. Run TWitness(A,R,d) and obtain
    $$w=\left(\left(j_{1},\ldots ,j_{l}\right)\in {\left\{0,1\right\}}^{l},\left(w_{l}\ldots ,w_{1}\right)\in {\left({\left\{0,1\right\}}^{nk}\right)}^{l}\right).$$
    Recall that *w* is a witness to the fact that d∈R.
  3. Sample a seed $s\leftarrow {\left\{0,1\right\}}^{n}$, generate a matrix $B=\mathrm{PRG}\left(s\right)\in Z_{q}^{n\times m}$ and compute b=B·xmodq. Then, produce an NIZKAoKΠ by repeating our interactive protocol κ=ω(logn) times. By using the Fiat-Shamir heuristic, we transform Π to the triple
    $$\Pi =\left({\left\{{\mathrm{CMT}}_{i}\right\}}_{i=1}^{\kappa },\mathrm{CH},{\left\{{\mathrm{RSP}}_{i}\right\}}_{i=1}^{\kappa }\right),$$
    where
    $$\mathrm{CH}=H_{\mathrm{FS}}\left(M,{\left\{{\mathrm{CMT}}_{i}\right\}}_{i=1}^{\kappa },A,B,u,b,R\right)=\left(Ch_{1},\ldots ,Ch_{\kappa }\right)\in {\left\{1,2,3\right\}}^{\kappa }.$$
  4. Output Σ=(s,b,Π).
- Verify(pp,R,M,Σ): Pm inputs pp,R,M,Σ, the verification procedure is detailed as follows:
  1. Run TAcc(A,R) and obtain u.
  2. Parse $\Sigma =\left(s,b,{\left\{{\mathrm{CMT}}_{i}\right\}}_{i=1}^{\kappa },\mathrm{CH},{\left\{{\mathrm{RSP}}_{i}\right\}}_{i=1}^{\kappa }\right)$. Let B=PRG(s). Output 0 if
    $$\left(Ch_{1},\ldots ,Ch_{\kappa }\right)\neq H_{\mathrm{FS}}\left(M,{\left\{{\mathrm{CMT}}_{i}\right\}}_{i=1}^{\kappa },A,B,u,b,R\right).$$
  3. For i=1,…,κ, check the validity of RSP${}_{i}$w.r.t. CMT${}_{i}$ and $Ch_{i}$. If all the conditions hold, output 1; otherwise, output 0.
- $\mathrm{EvidenceGen}(\mathrm{pp},R,sk_{i},\Sigma )$: On inputs pp, *R*, a secret key $sk_{i}=x^{\prime }$, and the pair (s,b) contained in Σ, the algorithm produces a piece of evidence $\xi_{i}$ as follows:
  1. Run TAcc(A,R) and obtain the Merkle tree’s root $u\in {\left\{0,1\right\}}^{nk}$.
  2. Let $pk_{i}=d^{\prime }=\mathrm{bin}\left(A\cdot x^{\prime }\;\;\;\mathrm{mod}\;q\right)$. Generate a witness
    $$w^{\prime }=\left(\left(j_{1}^{\prime },\ldots ,j_{l}^{\prime }\right)\in {\left\{0,1\right\}}^{l},\left(w_{l}^{\prime }\ldots ,w_{1}^{\prime }\right)\in {\left({\left\{0,1\right\}}^{nk}\right)}^{l}\right)$$
    to the fact that $d^{\prime }\in R$ by running $\mathrm{TWitness}(A,R,d^{\prime })$, i.e., $d^{\prime }$ was properly accumulated in u.
  3. Let B=PRG(s). Compute $b^{\prime }=B\cdot x^{\prime }\;\;\;\mathrm{mod}\;q$ and generate a NIZKAoK$\Pi^{\prime }$ as in the signing algorithm to demonstrate the possession of a valid pair $\left(pk_{i},sk_{i}\right)=\left(d^{\prime },x^{\prime }\right)$ such that $b^{\prime }=B\cdot x^{\prime }\;\;\;\mathrm{mod}\;q$ and that $d^{\prime }\in R$, i.e.,
    $$\Pi^{\prime }=\left({\left\{{\mathrm{CMT}}_{i}^{\prime }\right\}}_{i=1}^{\kappa },{\mathrm{CH}}^{\prime },{\left\{{\mathrm{RSP}}_{i}^{\prime }\right\}}_{i=1}^{\kappa }\right),$$
    where
    $${\mathrm{CH}}^{\prime }=H_{\mathrm{FS}}\left({\left\{{\mathrm{CMT}}_{i}^{\prime }\right\}}_{i=1}^{\kappa },A,B,u,b^{\prime },R\right)\in {\left\{1,2,3\right\}}^{\kappa }.$$
  4. Output $\xi_{i}=\left(s,b^{\prime },\Pi^{\prime }\right)$. Note that $\xi_{i}$ can be seen just as a signature on the empty message with the given seed *s* (instead of choosing a random seed by the algorithm itself).
- $\mathrm{EvidenceCheck}(\mathrm{pp},R,i,\xi_{i},\Sigma )$: On inputs $\mathrm{pp},R,i,\xi_{i},\Sigma$, the evidence $\xi_{i}$ is checked as follows:
  1. Check the validity of $\xi_{i}$ and Σ by verifying the underlying protocols. If either is invalid, then output “reject”.
  2. If $\left(s,b^{\prime }\right)=\left(s,b\right)$, then output “confirmation”; otherwise, output “disavowal”.

### Analysis of Our NDRS Scheme

We first briefly analyze the correctness and efficiency properties.

**Theorem** **3** (Correctness and Efficiency). *The NDRS scheme described in the previous section is correct and produces signatures of bit-size $\tilde{O}\left(n\cdot \log N\right)$.*

**Correctness.** It is easy to check that:

- By the perfect completeness of the argument system presented in the previous section, each member of a ring is always capable of obtaining a tuple (x,d,w) such that
  $$\left((A,B,u,b),d,w,x\right)\in R_{\mathrm{NDRS}}.$$
  Thus, by the Fiat-Shamir heuristic, the ring signature on *M* is valid.
- Meanwhile, for any signature Σ=(s,b,Π), the real signer can always produce a piece of valid evidence $\xi =(s,b^{\prime },\Pi^{\prime })$ such that $b=b^{\prime }$, i.e., EvidenceCheck outputs ‘confirmation’.
- By the randomness of the secret keys $x,x^{\prime }\leftarrow {\left\{0,1\right\}}^{m}$, the non-real signer can always produce a piece of valid evidence $\tilde{\xi }=\left(s,\tilde{b},\tilde{\Pi }\right)$ such that $b=B\cdot x\;\mathrm{mod}\;q\neq b^{\prime }=B\cdot x^{\prime }\;\mathrm{mod}\;q$ with overwhelming probability.

**Efficiency.** It is not hard to check that the underlying interactive procedure in previous section has communication cost $\tilde{O}\left(l\cdot n\right)$; therefore, the resulting signature has bit-size $\tilde{O}\left(\kappa \cdot l\cdot n+n\right)=\tilde{O}\left(n\cdot \log N\right)$.

Now, we analyze the security requirements: anonymity, traceability, and non-frameability.

**Theorem** **4** (Anonymity). *Assume that COM is a statistical hiding commitment scheme. Then, our NDRS scheme provides statistical anonymity in the random oracle model.*

**Proof.**  We consider a sequence of games. The challenger C runs experiment ${\mathrm{Exp}}_{NDRS,A}^{\mathrm{anon}-0}\left(n\right)$ in the first game, while, in the last one, it runs ${\mathrm{Exp}}_{NDRS,A}^{\mathrm{anon}-1}\left(n\right)$.

**Game G**${}_{0}^{\left(b\right)}$: Exactly, it is the real experiment ${\mathrm{Exp}}_{NDRS,A}^{\mathrm{anon}-b}\left(n\right)$, where the adversary is given a challenge signature $\Sigma^{*}\leftarrow \mathrm{Sign}\left(\mathrm{pp},\left\{pk_{i_{0}},pk_{i_{1}}\right\},sk_{i_{b}},M^{*}\right)$. Namely, given $\left(i_{0},i_{1},M^{*}\right)$, the challenger C chooses a random b←{0,1} and computes a legitimate signature $\Sigma^{*}$ using the secret key $sk_{i_{b}}=x_{i_{b}}$ of user $i_{b}$:
  1. Run TAcc(A,R) and obtain $u\in {\left\{0,1\right\}}^{nk}$, where $R=\{pk_{i_{0}},pk_{i_{1}}\}$.
  2. Run $\mathrm{TWitness}(A,R,d_{i_{b}})$ and obtain a witness $w_{i_{b}}$ to the fact that $d_{i_{b}}=A\cdot x_{i_{b}}\;\mathrm{mod}\;q\in R$.
  3. Sample a seed $s\leftarrow {\left\{0,1\right\}}^{n}$, generate matrix $B=\mathrm{PRG}\left(s\right)\in Z_{q}^{n\times m}$ and compute $b=B\cdot x_{i_{b}}\;\;\;\;\mathrm{mod}\;q$. Then, produce a NIZKAoKΠ with public input (A,B,u,b) and prover’s witness $\left(d_{i_{b}},w_{i_{b}},x_{i_{b}}\right)$, i.e.,
    $$\Pi =\left({\left\{{\mathrm{CMT}}_{i}\right\}}_{i=1}^{\kappa },\mathrm{CH},{\left\{{\mathrm{RSP}}_{i}\right\}}_{i=1}^{\kappa }\right),$$
    where
    $$\mathrm{CH}=H_{\mathrm{FS}}\left(M^{*},{\left\{{\mathrm{CMT}}_{i}\right\}}_{i=1}^{\kappa },A,B,u,b,R\right)\in {\left\{1,2,3\right\}}^{\kappa }.$$
  4. Output $\Sigma^{*}=\left(B,b,\Pi \right)$.
**Game G**${}_{1}$: Generally, this game is identical to G${}_{0}^{\left(b\right)}$, except that the challenge signature $\Sigma^{*}$ is made independent of the coin *b*, while preserving the statistical closeness to **G**${}_{0}^{\left(b\right)}$. In more detail, the following modifications are introduced with respect to **G**${}_{0}^{\left(b\right)}$:
  1. In Step 3, we change how the vector b is generated. Specifically, C samples $b\leftarrow Z_{q}^{n}$ uniformly at random, instead of computing $b=B\cdot x_{i_{b}}\;\;\;\mathrm{mod}\;q$.
  2. In addition, in Step 3, the proof Π contained in the challenge signature $\Sigma^{*}$ is produced in the simulation manner by C’s programming on the random oracle $H_{\mathrm{FS}}\left(\cdot \right)$.
    (a) For each j∈[κ], choose a ‘fake challenge’ ${\bar{Ch}}_{j}\leftarrow \left\{1,2,3\right\}$ and prepare the ‘commitment’ ${\mathrm{CMT}}_{j}$ according to ${\bar{Ch}}_{j}$. Then, randomly pick a ‘real challenge’ $Ch_{j}\leftarrow \left\{1,2,3\right\}\setminus \left\{{\bar{Ch}}_{j}\right\}$.
    (b) Program the random oracle and set
      $$\mathrm{CH}={\left\{Ch_{j}\right\}}_{j=1}^{\kappa }=H_{\mathrm{FS}}\left(M^{*},{\left\{{\mathrm{CMT}}_{j}\right\}}_{j=1}^{\kappa },A,B,u,b,R\right).$$
    (c) Prepare the ‘response’ ${\left\{{\mathrm{RSP}}_{j}\right\}}_{j=1}^{\kappa }$ in accordance with the normal procedure.
    (d) Output
      $$\Sigma^{*}=\left(s,b,\Pi \right)=\left(s,b,{\left\{{\mathrm{CMT}}_{i}\right\}}_{i=1}^{\kappa },\mathrm{CH},{\left\{{\mathrm{RSP}}_{i}\right\}}_{i=1}^{\kappa }\right).$$
  Observe that, for each j∈[κ], $Ch_{j}$ is uniformly distributed in {1,2,3}, satisfying the requirement on the output of the random oracle. Besides, ${\mathrm{CMT}}_{j}$ and ${\mathrm{RSP}}_{j}$ are prepared in the same way as in Lemma A2 for proving the zero-knowledge property, implying that the challenge signature is valid. Finally, notice that the vector b in this game or **G**${}_{0}^{\left(b\right)}$ follows a uniform distribution over $Z_{q}^{n}$. As a result, **G**${}_{0}^{\left(b\right)}$ and **G**${}_{1}$ are statistically indistinguishable.

Now, we obtain a sequence of indistinguishable games **G**${}_{0}^{\left(0\right)}$, **G**${}_{1}$ and **G**${}_{0}^{\left(1\right)}$. Since **G**${}_{1}$ is independent of the random coin *b*, the advantage of A in **G**${}_{1}$ is 0. Then, we have the advantage of A in **G**${}_{0}^{\left(0\right)}$ and **G**${}_{0}^{\left(1\right)}$ is negligible. This completes the proof. □

Next, we prove the traceability and the non-frameability. Before doing so, we first recall two useful lemmas.

**Lemma** **1** (Reference [8]). *For any matrix $A\in Z_{q}^{n\times m}$ and a uniform random $x\in {\left\{0,1\right\}}^{m}$, the probability that there exists another $x^{\prime }\in {\left\{0,1\right\}}^{m}\setminus \left\{x\right\}$ such that $A\cdot x=A\cdot x^{\prime }\;\;\;\mathrm{mod}\;q$ is at least $1-2^{n\cdot \log q-m}$.*

**Lemma** **2** (Reference [13]). *Let SS be a signature scheme with security parameter n. Let A be a PPT algorithm whose input consists only of public data and which can ask $q_{H}>0$ queries to the random oracle. Assume that A produces within time bound T a valid signature $\left({\left\{{\mathrm{CMT}}_{i}\right\}}_{i=1}^{\kappa },\mathrm{CH},{\left\{{\mathrm{RSP}}_{i}\right\}}_{i=1}^{\kappa }\right)$ of message M with probability ϵ. Then, within time $32\cdot T\cdot q_{H}/\epsilon$ and with probability $\epsilon^{\prime }>1/2$, a replay of A outputs 3 valid signatures of M:*

$$\begin{array}{c} \left({\left\{{\mathrm{CMT}}_{i}\right\}}_{i=1}^{\kappa },{\mathrm{CH}}^{\left(1\right)},{\left\{{\mathrm{RSP}}_{i}^{\left(1\right)}\right\}}_{i=1}^{\kappa }\right),\;\;\;\left({\left\{{\mathrm{CMT}}_{i}\right\}}_{i=1}^{\kappa },{\mathrm{CH}}^{\left(2\right)},{\left\{{\mathrm{RSP}}_{i}^{\left(2\right)}\right\}}_{i=1}^{\kappa }\right),\\ \left({\left\{{\mathrm{CMT}}_{i}\right\}}_{i=1}^{\kappa },{\mathrm{CH}}^{\left(3\right)},{\left\{{\mathrm{RSP}}_{i}^{\left(3\right)}\right\}}_{i=1}^{\kappa }\right) \end{array}$$

*for the same ${\left\{{\mathrm{CMT}}_{i}\right\}}_{i=1}^{\kappa }$ such that ${\mathrm{CH}}^{\left(1\right)},{\mathrm{CH}}^{\left(2\right)},{\mathrm{CH}}^{\left(3\right)}$ are pairwise distinct.*

**Theorem** **5** (Traceability and Non-frameability). *Our NDRS scheme provides traceability and non-frameability in the random oracle model if the ${\mathrm{SIVP}}_{\tilde{O}\left(n\right)}$ is hard.*

**Proof.**  Assume that there exists a PPT A has nonnegligible advantage ϵ in the experiment **Exp**${}_{NDRS,A}^{\mathrm{trace}}\left(n\right)$ or **Exp**${}_{NDRS,A}^{\mathrm{nf}}\left(n\right)$, i.e., A is able to output a valid signature $\Sigma^{*}$ on message $M^{*}$ under some ring $R^{*}=\left(pk_{i_{0}},\ldots ,pk_{i_{r}}\right)=\left(d_{0},\ldots ,d_{r}\right)$ such that

- either $\mathrm{EvidenceCheck}(\mathrm{pp},R^{*},i_{j},\xi_{i_{j}},\Sigma^{*})$ will output ‘disavowal’ for each j∈{0,⋯,r}, where $\xi_{i_{j}}$ is a piece of evidence generated by user $i_{j}$;
- or $\mathrm{EvidenceCheck}(\mathrm{pp},R^{*},i_{j^{*}},\xi_{i_{j^{*}}},\Sigma^{*})$ will output ‘confirmation’ for some honest user $i_{j^{*}}$.

We construct an algorithm B that solves the ${\mathrm{SIVP}}_{\tilde{O}\left(n\right)}$ problem with nonnegligible probability. Let pp=A. During the game, B generates the secrets of all the queried users as in the real scheme. With these secret keys, B is capable of faithfully answering all the queries. For the random oracle $H_{\mathrm{FS}}\left(\cdot \right)$, we assume without loss of generality that: (1) A makes any given query to $H_{\mathrm{FS}}\left(\cdot \right)$ only once; (2) if A outputs a signature, then A had previously queried $H_{\mathrm{FS}}\left(\cdot \right)$.

When A halts, it outputs a valid triple $\left(R^{*},M^{*},\Sigma^{*}\right)$, where

$$\Sigma^{*}=\left(s^{*},b^{*},{\left\{{\mathrm{CMT}}_{i}^{*}\right\}}_{i=1}^{\kappa },{\mathrm{CH}}^{*},{\left\{{\mathrm{RSP}}_{i}^{*}\right\}}_{i=1}^{\kappa }\right).$$

We denote by $q_{H}$ the upper bound on the number of queries that A makes to $H_{\mathrm{FS}}\left(\cdot \right)$.

Then, by Lemma 2, when B runs up to $32\cdot q_{H}/\epsilon$ extra executions of A with the same random tap and inputs as in the first execution, with probability at least 1/2, A will get a 3-fork responses ${\mathrm{CH}}^{\left(1\right)},{\mathrm{CH}}^{\left(2\right)},{\mathrm{CH}}^{\left(3\right)}$ (pairwise distinct) from the oracle $H_{\mathrm{FS}}\left(\cdot \right)$.

With probability $1-{\left(7/9\right)}^{\kappa }$, there exists some j∈[κ] for which the *j*-th bits of ${\mathrm{CH}}^{\left(1\right)},{\mathrm{CH}}^{\left(2\right)}$, and ${\mathrm{CH}}^{\left(3\right)}$ are $\left\{Ch_{j}^{\left(1\right)},Ch_{j}^{\left(2\right)},Ch_{j}^{\left(3\right)}\right\}=\left\{1,2,3\right\}$. By the soundness of the argument system for the relation $R_{\mathrm{NDRS}}$, B is able to extract a tuple $\left(d^{*},w^{*},x^{*}\right)$ from the responses ${\mathrm{RSP}}_{j}^{\left(1\right)},{\mathrm{RSP}}_{j}^{\left(2\right)},{\mathrm{RSP}}_{j}^{\left(3\right)}$ such that

$$A\cdot x^{*}=G\cdot d^{*}\;\;\;\mathrm{mod}\;q,\;\;\;\;\mathrm{TVerify}\left(A,u^{*},d^{*},w^{*}\right)=1.$$

According to the value of $d^{*}$, there are two cases:

- $d^{*}\notin R^{*}=\left(d_{0},\ldots ,d_{r}\right)$. This means B can use $\left(R^{*},d^{*},w^{*}\right)$ to break the security of the underlying accumulator, whose security is based on the assumption that ${\mathrm{SIVP}}_{\tilde{O}\left(n\right)}$ is hard [8].
- $d^{*}\in R^{*}=\left(d_{0},\ldots ,d_{r}\right)$, i.e., $d^{*}=d_{j^{*}}$. Note that the secret key $sk_{i_{j^{*}}}$ consists of a vector $x_{i_{j^{*}}}\in {\left\{0,1\right\}}^{m}$ such that $A\cdot x_{i_{j^{*}}}=G\cdot d_{j^{*}}\;\;\;\mathrm{mod}\;q$. If $x_{i_{j^{*}}}\neq x^{*}$, then $x_{i_{j^{*}}}-x^{*}\in {\left\{-1,0,1\right\}}^{m}$ is a valid solution for the ${\mathrm{SIS}}_{n,m,q,1}$ instance A.
  According to the experiments with respect to traceability and non-frameability, we distinguish the following two cases to discuss the probability that $x_{i_{j^{*}}}\neq x^{*}$.
  - In the experiment **Exp**${}_{NDRS,A}^{\mathrm{trace}}\left(n\right)$, A has corrupted user $i_{j^{*}}$, acts as the real malicious signer, and manages to evade the traceability. We claim that $x_{i_{j^{*}}}\neq x^{*}$, since A will otherwise be detected as the real singer by the algorithm EvidenceCheck$\left(\mathrm{pp},R^{*},i_{j^{*}},\xi_{i_{j^{*}}},\Sigma \right)$, where $\xi_{i_{j^{*}}}$ contains an element $b=B^{*}\cdot x_{i_{j^{*}}}\;\;\;\mathrm{mod}\;q$.
  - In the experiment **Exp**${}_{NDRS,A}^{\mathrm{nf}}\left(n\right)$, A did not corrupt user $i_{j^{*}}$, and temps to produce a valid signature such that the target victim $i_{j^{*}}$ will be detected as the real signer. We claim that $x_{i_{j^{*}}}\neq x^{*}$ with probability greater than 1/2 by the following two facts: (1) There exists another vector $x^{*}\in {\left\{0,1\right\}}^{m}$ such that $A\cdot x^{*}=A\cdot x_{i_{j^{*}}}\;\;\;\mathrm{mod}\;q$ by Lemma 1. (2) The underlying argument system is zero-knowledge, which implies witness indistinguishability; thus, A can hardly get useful information from the signing queries.

In conclusion, in the experiment **Exp**${}_{NDRS,A}^{\mathrm{trace}}\left(n\right)$ or **Exp**${}_{NDRS,A}^{\mathrm{nf}}\left(n\right)$, a successful attacker A implies an attacker B that either defeats the soundness of the argument system, or breaks the security of the accumulator, or directly solves an ${\mathrm{SIS}}_{n,m,q,1}^{\infty }$ instance A. Thus, our scheme provides traceability and non-frameability in the random oracle model, assuming that the ${\mathrm{SIVP}}_{\tilde{O}\left(n\right)}$ problem is hard. □

## 5. Conclusions

In this work, we propose an efficient lattice-based NDRS scheme by using the techniques developed in Reference [8]. Our scheme has signature size only logarithmic to the ring size, and we prove its security in the random oracle model under the SIS assumption. Notice that, in our NDRS scheme, each secret key can only be used, at most, k−1 times for producing ring signatures, where k=logq; otherwise, the secret key will be figured out from B’s and corresponding b’s. The direct way to increase the number of ring signatures for each user is to increase the parameter *q*, which will reduce efficiency. A better way is to develop new techniques that is able to authenticate the user’s identity while producing the ring signature for relative small *q*. We leave it as a future work.

## Appendix A. Proof of Theorem 2

Our proof follows closely from that of Lemma 4 in Reference [8].

**Completeness and Efficiency.** It can be checked that the protocol has perfect completeness: if P is honest and follows the protocol, then V always outputs 1. The communication cost of the protocol is of order $\tilde{O}\left(l\cdot m\cdot \log q\right)=\tilde{O}\left(l\cdot n\right)$.

**Lemma** **A1** (Zero-Knowledge Property). *Assume that COM is a statistical hiding commitment scheme, then the protocol is a statistical zero-knowledge proof.*

**Proof.** To show the zero-knowledge property, we construct an efficient simulator S that outputs a simulated transcript statistically indistinguishable from the one produced by the honest prover. The simulator S first randomly samples $\bar{Ch}\leftarrow \left\{1,2,3\right\}$, which serves as a prediction of the challenge value that $\hat{V}$ will not choose.

Ch¯=1 First, S computes $x^{\prime }\in Z_{q}^{2m},\;v_{1}^{\prime },\ldots ,v_{l}^{\prime }\in Z_{q}^{m}$ and $z_{1}^{\prime },\ldots ,z_{l}^{\prime },\;\;y_{1}^{\prime },\ldots ,y_{l}^{\prime }\in Z_{q}^{2m}$ such that
  $$\begin{array}{cc} \hat{A}\cdot x^{\prime } & =G^{*}\cdot v_{l}^{\prime }\;\;\;\mathrm{mod}\;q;\\ \hat{B}\cdot x^{\prime } & =b\;\;\;\mathrm{mod}\;q;\\ A^{*}\cdot z_{1}^{\prime }+A^{*}\cdot y_{1}^{\prime } & =G\cdot u\;\;\;\mathrm{mod}\;q;\\ A^{*}\cdot z_{i+1}^{\prime }+A^{*}\cdot y_{i+1}^{\prime } & =G^{*}\cdot v_{i}^{\prime }\;\;\;\mathrm{mod}\;q\;\;\forall \;i\in \left[l-1\right]. \end{array}$$
  Then, sample randomness $\rho_{1},\rho_{2},\rho_{3}$ for COM and
  $$\begin{array}{cc} \; & \tau \leftarrow S_{2m};\;b_{1},\ldots ,b_{l}\leftarrow \left\{0,1\right\};\;\pi_{1},\ldots ,\pi_{l},\;\phi_{1},\ldots ,\phi_{l}\leftarrow S_{m}\\ \; & r_{x};\;r_{v_{1}},\ldots ,r_{v_{l}}\leftarrow Z_{q}^{m};\;r_{z_{1}},\ldots ,r_{z_{l}},\;r_{y_{1}},\ldots ,r_{y_{l}}\leftarrow Z_{q}^{2m}. \end{array}$$
  Finally, send to V the commitment CMT=$\left(C_{1}^{\prime },C_{2}^{\prime },C_{3}^{\prime }\right)$, where
  $$\begin{array}{cc} C_{1}^{\prime } & =\mathrm{COM}(\tau ;\;\hat{A}\cdot r_{x}-G^{*}\cdot r_{v_{l}};\;\hat{B}\cdot r_{x};\;{\left\{b_{i},\pi_{i},\phi_{i}\right\}}_{i=1}^{l};\;A^{*}\cdot r_{z_{1}}+A^{*}\cdot r_{y_{1}};\\ \; & \;\;\;\;\;\;\;\;\;\;\;\;\;\;{\left\{A^{*}\cdot r_{z_{i+1}}+A^{*}\cdot r_{y_{i+1}}-G^{*}\cdot r_{v_{i}}\right\}}_{i=1}^{l-1};\;\rho_{1})\\ C_{2}^{\prime } & =\mathrm{COM}\left(\tau \left(r_{x}\right);\;{\left\{\pi_{i}\left(r_{v_{i}}\right),F_{b_{i},\pi_{i}}\left(r_{z_{i}}\right),F_{{\bar{b}}_{i},\phi_{i}}\left(r_{y_{i}}\right)\right\}}_{i=1}^{l};\;\rho_{2}\right)\\ C_{3}^{\prime } & =\mathrm{COM}\left(\tau \left(x^{\prime }+r_{x}\right);\;{\left\{\pi_{i}\left(v_{i}^{\prime }+r_{v_{i}}\right),F_{b_{i},\pi_{i}}\left(z_{i}^{\prime }+r_{z_{i}}\right),F_{{\bar{b}}_{i},\phi_{i}}\left(y_{i}^{\prime }+r_{y_{i}}\right)\right\}}_{i=1}^{l};\;\rho_{3}\right). \end{array}$$
  After receiving Ch from $\hat{V}$, $\hat{S}$ responds as follows:
  - If Ch=1: Output ⊥ and abort.
  - If Ch=2: Send
    $$\mathrm{RSP}=\left(\tau ;\;x^{\prime }+r_{x};\;{\left\{b_{i},\pi_{i},\phi_{i},v_{i}^{\prime }+r_{v_{i}},z_{i}^{\prime }+r_{z_{i}},y^{\prime }r_{y_{i}}\right\}}_{i=1}^{l};\;\rho_{1};\;\rho_{3}\right).$$
  - If Ch=3: Send
    $$\mathrm{RSP}=\left(\tau ;\;r_{x};\;{\left\{b_{i},\pi_{i},\;\phi_{i},r_{v_{i}},r_{z_{i}},r_{y_{i}}\right\}}_{i=1}^{l};\;\rho_{1};\;\rho_{2}\right).$$
Ch¯=2 First, S samples
  $$\begin{array}{cc} \; & x^{\prime }\leftarrow B_{2m}^{m};\;j_{1}^{\prime },\ldots ,j_{l}^{\prime }\leftarrow \left\{0,1\right\};\;v_{1}^{\prime },\ldots ,v_{l}^{\prime };\;w_{1}^{\prime },\ldots ,w_{l}^{\prime }\leftarrow B_{m}^{nk};\\ \; & \tau \leftarrow S_{2m};\;b_{1},\ldots ,b_{l}\leftarrow \left\{0,1\right\};\;\pi_{1},\ldots ,\pi_{l},\;\phi_{1},\ldots ,\phi_{l}\leftarrow S_{m}\\ \; & r_{x};\;r_{v_{1}},\ldots ,r_{v_{l}}\leftarrow Z_{q}^{m};\;r_{z_{1}},\ldots ,r_{z_{l}},\;r_{y_{1}},\ldots ,r_{y_{l}}\leftarrow Z_{q}^{2m}. \end{array}$$
  Then, compute $z_{i}^{\prime }=\mathrm{ext}\left(j_{i}^{\prime },v_{i}^{\prime }\right),\;y_{i}^{\prime }=\mathrm{ext}\left({\bar{j}}_{i}^{\prime },w_{i}^{\prime }\right)$ for each j∈[l]. Finally, send the commitment CMT computed as in case $\bar{Ch}=1$.
  After receiving Ch from $\hat{V}$, $\hat{S}$ responds as follows.
  - If Ch=1: Send
    $$\mathrm{RSP}=\left(\tau \left(x^{\prime }\right);\;\tau \left(r_{x}\right);\;{\left\{j_{i}^{\prime }⊕b_{i},\pi_{i}\left(v_{i}^{\prime }\right),\phi_{i}\left(w_{i}^{\prime }\right),\pi_{i}\left(r_{v_{i}}\right),F_{b_{i},\pi_{i}}\left(\}\}r_{z_{i}}\right),F_{{\bar{b}}_{i},\phi_{i}}\left(r_{y_{i}}\right)\right\}}_{i=1}^{l};\;\rho_{1};\;\rho_{3}\right).$$
  - If Ch=2: Output ⊥ and abort.
  - If Ch=3: Send RSP computed in the same manner as in the case ($\bar{Ch}=1,Ch=3$).
Ch¯=3 : First, S sample randomness as in the case $\bar{Ch}=2$. Then, send the commitments CMT=$\left(C_{1}^{\prime },C_{2}^{\prime },C_{3}^{\prime }\right)$, where $C_{2}^{\prime },C_{3}^{\prime }$ are computed as in $\bar{Ch}=1$, and $C_{1}^{\prime }$ is computed as
  $$\begin{array}{c} C_{1}^{\prime }=\mathrm{COM}(\tau ;\;\hat{A}\cdot \left(x^{\prime }+r_{x}\right)-G^{*}\cdot \left(v_{l}^{\prime }+r_{v_{l}}\right);\;\hat{B}\cdot \left(x^{\prime }+r_{x}\right);\;{\left\{b_{i},\pi_{i},\phi_{i}\right\}}_{i=1}^{l};\\ A^{*}\cdot \left(z_{1}^{\prime }+r_{z_{1}}\right)+A^{*}\cdot \left(y_{1}^{\prime }+r_{y_{1}}\right)-G\cdot u;\\ {\left\{A^{*}\cdot \left(z_{i+1}^{\prime }+r_{z_{i+1}}\right)+A^{*}\cdot \left(y_{i+1}^{\prime }+r_{y_{i+1}}\right)-G^{*}\cdot \left(v_{i}^{\prime }+r_{v_{i}}\right)\right\}}_{i=1}^{l-1};\;\rho_{1}). \end{array}$$
  After receiving Ch from $\hat{V}$, $\hat{S}$ responds as follows.
  - If Ch=1: Send RSP computed as in the case ($\bar{Ch}=2,Ch=1$).
  - If Ch=2: Send RSP computed as in the case ($\bar{Ch}=1,Ch=2$).
  - If Ch=3: Output ⊥ and abort.

Because COM is statistically hiding, we have that, whenever S does not halt, it will output an accepting transcript, whose distribution is statistically close to that of the real prover. Besides, S halts with probability 1/3. Therefore, S can successfully emulate the honest prover with probability 2/3. □

To show the argument of knowledge property, it is enough to show that the protocol has the special soundness property [24].

**Lemma** **A2** (Argument of Knowledge Property). *Assume that COM is a statistical hiding commitment scheme, and then there exists an efficient knowledge extractor K that, given 3 valid responses $\left({\mathrm{RSP}}_{1},{\mathrm{RSP}}_{2},{\mathrm{RSP}}_{3}\right)$ to the same commitment CMT, outputs a triple $\left(d^{\prime },w^{\prime },x^{\prime }\right)$ such that $\left(\left(A,B,u,b\right);d^{\prime },w^{\prime },x^{\prime }\right)\in R_{\mathrm{NDRS}}$.*

**Proof.** Denote the 3 valid responses $\left({\mathrm{RSP}}_{1},{\mathrm{RSP}}_{2},{\mathrm{RSP}}_{3}\right)$ to the same commitment CMT as follows:

$$\begin{array}{cc} {\mathrm{RSP}}_{1} & =\left({\tilde{x}}^{*};\;{\tilde{r}}_{x};\;{\left\{{\tilde{b}}_{i},{\tilde{v}}_{i}^{*},{\tilde{w}}_{i}^{*},{\tilde{r}}_{v_{i}},{\tilde{r}}_{z_{i}},{\tilde{r}}_{y_{i}}\right\}}_{i=1}^{l};\;\rho_{2};\;\rho_{3}\right),\\ {\mathrm{RSP}}_{2} & =\left(\tau^{\prime };\;s_{x};\;{\left\{b_{i}^{\prime },\pi_{i}^{\prime },\phi_{i}^{\prime },s_{v_{i}},s_{z_{i}},s_{y_{i}}\right\}}_{i=1}^{l};\;\rho_{1};\;\rho_{3}\right),\\ {\mathrm{RSP}}_{3} & =\left(\tau^{\prime \prime };\;r_{x}^{\prime };\;{\left\{b_{i}^{\prime \prime },\pi_{i}^{\prime \prime },\;\phi_{i}^{\prime \prime },r_{v_{i}}^{\prime },r_{z_{i}}^{\prime },r_{y_{i}}^{\prime }\right\}}_{i=1}^{l};\;\rho_{1};\;\rho_{2}\right). \end{array}$$

The validity of RSP${}_{1}$ implies that ${\tilde{x}}^{*}\in B_{2m}^{m}$ and $\forall \;i\in \left[l\right]:\;{\tilde{v}}_{i}^{*},\;{\tilde{w}}_{i}^{*}\in B_{m}^{nk}$. Besides, we have:

$$\begin{array}{cc} \; & \tau^{\prime }=\tau^{\prime \prime };\;\tau^{\prime \prime }\left(r_{x}^{\prime }\right)={\tilde{r}}_{x};\;\tau^{\prime }\left(s_{x}\right)={\tilde{x}}^{*}+{\tilde{r}}_{x},\\ \; & \hat{A}\cdot s_{x}-G^{*}\cdot s_{v_{l}}=\hat{A}\cdot r_{x}^{\prime }-G^{*}\cdot r_{v_{l}^{\prime }}\;\;\;\mathrm{mod}\;q,\\ \; & \hat{B}\cdot s_{x}=b+\hat{B}\cdot r_{x}^{\prime }\;\;\;\mathrm{mod}\;q,\\ \; & A^{*}\cdot s_{z_{1}}+A^{*}\cdot s_{y_{1}}-G\cdot u=A^{*}\cdot r_{z_{1}}^{\prime }+A^{*}\cdot r_{y_{1}}^{\prime }\;\;\;\mathrm{mod}\;q, \end{array}$$

and, for each i∈[l−1]:

$$A^{*}\cdot s_{z_{i+1}}+A^{*}\cdot s_{y_{i+1}}-G^{*}\cdot s_{v_{i}}=A^{*}\cdot r_{z_{i+1}}^{\prime }+A^{*}\cdot r_{y_{i+1}}^{\prime }-G^{*}\cdot r_{v_{i}}^{\prime }\;\;\;\mathrm{mod}\;q,$$

and, for all i∈[l]:

$$\begin{array}{cc} \; & b_{i}^{\prime }=b_{i}^{\prime \prime };\;\pi^{\prime }=\pi^{\prime \prime };\;\phi^{\prime }=\phi^{\prime \prime },\\ \; & \pi_{i}^{\prime }\left(s_{x}\right)={\tilde{x}}^{*}+{\tilde{r}}_{x};\;\pi_{i}^{\prime \prime }\left(r_{v}^{\prime }\right)={\tilde{r}}_{v_{i}},\\ \; & F_{b_{i}^{\prime },\pi_{i}^{\prime }}\left(s_{z_{i}}\right)=\mathrm{ext}\left({\tilde{b}}_{i},{\tilde{v}}_{i}^{*}\right)+{\tilde{r}}_{z_{i}};\;F_{b_{i}^{\prime \prime },\pi_{i}^{\prime \prime }}\left(r_{z_{i}}^{\prime }\right)={\tilde{r}}_{z_{i}},\\ \; & F_{{\bar{b}}_{i}^{\prime },\phi_{i}^{\prime }}\left(s_{y_{i}}\right)=\mathrm{ext}\left({\tilde{b}}_{i},{\tilde{w}}_{i}^{*}\right)+{\tilde{r}}_{y_{i}};\;F_{{\bar{b}}_{i}^{\prime \prime },\phi_{i}^{\prime \prime }}\left(r_{y_{i}}^{\prime }\right)={\tilde{r}}_{y_{i}}. \end{array}$$

Now, the knowledge extractor K takes the following steps to extract the secret. First, let $x^{*}=\tau^{\prime -1}\left({\tilde{x}}^{*}\right)$, and, for each i∈[l], let

$$j_{i}={\tilde{b}}_{i}⊕b_{i}^{\prime };\;v_{i}^{*}=\pi_{i}^{\prime -1}\left({\tilde{v}}_{i}^{*}\right);\;w_{i}^{*}=\phi_{i}^{\prime -1}\left({\tilde{w}}_{i}^{*}\right);\;z_{i}=s_{z_{i}}-r_{z_{i}}^{\prime };\;y_{i}=s_{y_{i}}-r_{y_{i}}^{\prime }.$$

Note that $x^{*}\in B_{2m}^{m}$, and, for each $i\in \left[l\right],\;v_{i}^{*},\;w_{i}^{*}\in B_{m}^{nk}$. Besides,

- $F_{b_{i}^{\prime },\pi_{i}^{\prime }}\left(z_{i}\right)=\mathrm{ext}\left({\tilde{b}}_{i},{\tilde{v}}_{i}^{*}\right)=\mathrm{ext}\left(j_{i}⊕b_{i}^{\prime },\pi^{\prime }\left(v_{i}^{*}\right)\right)\Leftrightarrow z_{i}=\mathrm{ext}\left(j_{i},v_{i}^{*}\right)$,
- $F_{{\bar{b}}_{i}^{\prime },\phi_{i}^{\prime }}\left(y_{i}\right)=\mathrm{ext}\left({\tilde{b}}_{i},{\tilde{w}}_{i}^{*}\right)=\mathrm{ext}\left({\bar{j}}_{i}⊕b_{i}^{\prime },\phi^{\prime }\left(w_{i}^{*}\right)\right)\Leftrightarrow y_{i}=\mathrm{ext}\left({\bar{j}}_{i},w_{i}^{*}\right)$.

Further more, $\hat{A}\cdot x^{*}=G^{*}\cdot v_{l}^{*}\;\;\;\mathrm{mod}\;q,\;\hat{B}\cdot x^{*}=b\;\;\;\mathrm{mod}\;q$ and

$$\begin{array}{cc} A^{*}\cdot z_{1}+A^{*}\cdot y_{1} & =G\cdot u\;\;\;\mathrm{mod}\;q,\\ A^{*}\cdot z_{i+1}+A^{*}\cdot y_{i+1} & =G^{*}\cdot v_{i}^{*}\;\;\;\mathrm{mod}\;q\;\;\forall \;i\in \left[l-1\right], \end{array}$$

which are equivalent to

$$\begin{array}{cc} A^{*}\cdot \mathrm{ext}\left(j_{1},v_{1}^{*}\right)+A^{*}\cdot \mathrm{ext}\left({\bar{j}}_{1},w_{1}^{*}\right) & =G\cdot u\;\;\;\mathrm{mod}\;q,\\ A^{*}\cdot \mathrm{ext}\left(j_{i+1},v_{i+1}^{*}\right)+A^{*}\cdot \mathrm{ext}\left({\bar{j}}_{i+1},w_{i+1}^{*}\right) & =G^{*}\cdot v_{i}^{*}\;\;\;\mathrm{mod}\;q\;\;\forall \;i\in \left[l-1\right]. \end{array}$$

Then, K drops the last *m* coordinates from $x^{*}$ and obtains $x^{\prime }\in {\left\{0,1\right\}}^{m}$. In addition, by dropping the last nk coordinates of $v_{1}^{*},\ldots ,v_{l}^{*},\;w_{1}^{*},\ldots ,w_{l}^{*}$, it obtains $v_{1}^{\prime },\ldots ,v_{l}^{\prime },\;w_{1}^{\prime },\ldots ,w_{l}^{\prime }\in {\left\{0,1\right\}}^{nk}$. Observe that $A\cdot x^{\prime }=G\cdot v_{l}^{\prime }\;\;\;\mathrm{mod}\;q,\;B\cdot x^{\prime }=b\;\;\;\mathrm{mod}\;q$. Thus, the following relations holds:

$$\begin{array}{cc} A\cdot \mathrm{ext}\left(j_{1},v_{1}^{\prime }\right)+A\cdot \mathrm{ext}\left({\bar{j}}_{1},w_{1}^{\prime }\right) & =G\cdot u\;\;\;\mathrm{mod}\;q,\\ A\cdot \mathrm{ext}\left(j_{i+1},v_{i+1}^{\prime }\right)+A\cdot \mathrm{ext}\left({\bar{j}}_{i+1},w_{i+1}^{\prime }\right) & =G\cdot v_{i}^{\prime }\;\;\;\mathrm{mod}\;q\;\;\forall \;i\in \left[l-1\right], \end{array}$$

which are equivalent to

$$\begin{array}{cc} \; & v_{0}^{\prime }=u,\\ \; & v_{i}^{\prime }={\bar{j}}_{i+1}\cdot h_{A}\left(v_{i+1}^{\prime },w_{i+1}^{\prime }\right)+j_{i+1}\cdot h_{A}\left(w_{i+1}^{\prime },v_{i+1}^{\prime }\right). \end{array}$$

Finally, let $d^{\prime }=v_{l}^{\prime }$ and $w^{\prime }=\left(\left(j_{1},\ldots ,j_{l}\right),\left(w_{l}^{\prime },\ldots ,w_{1}^{\prime }\right)\right)$, then $\mathrm{Verify}(A,u,d^{\prime },w^{\prime })=1$, and output $d^{\prime },w^{\prime },x^{\prime }$, which satisfy

$$\left(\left(A,B,u,d\right),d^{\prime },w^{\prime },x^{\prime }\right)\in R_{\mathrm{NDRS}}.$$

This concludes the proof. □

## References

[1] Rivest R.L., Shamir A., Tauman Y. (2006). How to leak a secret: Theory and applications of ring signatures. Theor. Comput. Sci..

[2] Naor M. (2002). Deniable ring authentication. CRYPTO 2002, Proceedings of the Annual International Cryptology Conference, Santa Barbara, CA, USA, 18–22 August 2002.

[3] Dodis Y., Kiayias A., Nicolosi A., Shoup V. (2004). Anonymous identification in *Ad Hoc Groups*. EUROCRYPT 2004, Proceedings of the Theory and Applications of Cryptographic Techniques, Interlaken, Switzerland, 2–6 May 2004.

[4] Chaum D., van Heyst E. (1991). Group signatures. EUROCRYPT 1991, Proceedings of the Workshop on the Theory and Application of of Cryptographic Techniques, Brighton, UK, 8–11 April 1991.

[5] Bellare M., Micciancio D., Warinschi B. (2003). Foundations of group signatures: Formal definitions, simplified requirements, and a construction based on general assumptions. EUROCRYPT 2003, Proceedings of the International Conference on the Theory and Applications of Cryptographic Techniques, Warsaw, Poland, 4–8 May 2003.

[6] Boyen X., Waters B. (2006). Compact group signatures without random oracles. EUROCRYPT 2006, Proceedings of the Annual International Conference on the Theory and Applications of Cryptographic Techniques, St. Petersburg, Russia, 28 May–1 June 2006.

[7] Groth J. (2007). Fully anonymous group signatures without random oracles. ASIACRYPT 2007, Proceedings of the International Conference on the Theory and Application of Cryptology and Information Security, Kuching, Malaysia, 2–6 December 2007.

[8] Libert B., Ling S., Nguyen K., Wang H. (2016). Zero-knowledge arguments for lattice-based accumulators: Logarithmic-size ring signatures and group signatures without trapdoors. EUROCRYPT 2016, Proceedings of the Annual International Conference on the Theory and Applications of Cryptographic Techniques, Vienna, Austria, 8–12 May 2016.

[9] Ling S., Nguyen K., Wang H., Xu Y. (2019). Lattice-based group signatures: Achieving full dynamicity (and deniability) with ease. Theor. Comput. Sci..

[10] Komano Y., Ohta K., Shimbo A., Kawamura S. (2006). Toward the fair anonymous signatures: Deniable ring signatures. CT-RSA 2006, Proceedings of the Cryptographers’ Track at the RSA Conference, San Jose, CA, USA, 13–17 February 2006.

[11] Shor P.W. (1997). Polynomial-time algorithms for prime factorization and discrete logarithms on a quantum computer. SIAM J. Comput.

[12] Gao W., Chen L., Hu Y., Newton C.J., Wang B., Chen J. (2019). Lattice-based deniable ring signatures. Int. J. Inf. Secur..

[13] Cheng S., Nguyen K., Wang H. (2016). Policy-based signature scheme from lattices. Des. Codes Cryptogr..

[14] Libert B., Ling S., Nguyen K., Wang H. (2017). Zero-knowledge arguments for lattice-based PRFs and applications to e-cash. ASIACRYPT 2017, Proceedings of the International Conference on the Theory and Application of Cryptology and Information Security, Hong Kong, China, 3–7 December 2017.

[15] Stern J. (1996). A new paradigm for public key identification. IEEE Trans. Inf. Theory.

[16] Jia H., Tang C. (2020). Cryptanalysis of a non-interactive deniable ring signature scheme. Int. J. Inf. Secur..

[17] Ajtai M. (2004). Generating hard instances of lattice problems. Quad. Mat..

[18] Micciancio D., Regev O. (2007). Worst-case to average-case reductions based on Gaussian measure. SIAM J. Comput..

[19] Gentry C., Peikert C., Vaikuntanathan V. (2008). Trapdoors for hard lattices and new cryptographic constructions. STOC 2008, Proceedings of the Fortieth Annual ACM Symposium on Theory of Computing, Victoria, BC, Canada, 17–20 May 2008.

[20] Micciancio D., Peikert C. (2013). Hardness of SIS and LWE with small parameters. CRYPTO 2013, Proceedings of the Annual Cryptology Conference, Santa Barbara, CA, USA, 18–22 August 2013.

[21] Jain A., Krenn S., Pietrzak K., Tentes A. (2012). Commitments and efficient zero-knowledge proofs from learning parity with noise. ASIACRYPT 2012, Proceedings of the International Conference on the Theory and Application of Cryptology and Information Security, Beijing, China, 2–6 December 2012.

[22] Benhamouda F., Camenisch J., Krenn S., Lyubashevsky V., Neven G. (2014). Better zero-knowledge proofs for lattice encryption and their application to group signatures. ASIACRYPT 2014, Proceedings of the International Conference on the Theory and Application of Cryptology and Information Security, Kaoshiung, Taiwan, 7–11 December 2014.

[23] Kawachi A., Tanaka K., Xagawa K. (2008). Concurrently secure identification schemes based on the worst-case hardness of lattice problems. ASIACRYPT 2008, Proceedings of the International Conference on the Theory and Application of Cryptology and Information Security, Melbourne, VIC, Australia, 7–11 December 2008.

[24] Groth J. (2004). Evaluating security of voting schemes in the universal composability framework. ACNS 2004, Proceedings of the InInternational Conference on Applied Cryptography and Network Security, Yellow Mountains, China, 8–11 June 2004.

